# The Influential Roles of Antibiotics Prophylaxis in Cirrhotic Patients with Peptic Ulcer Bleeding after Initial Endoscopic Treatments

**DOI:** 10.1371/journal.pone.0096394

**Published:** 2014-05-02

**Authors:** Shih-Cheng Yang, Jen-Chieh Chen, Wei-Chen Tai, Cheng-Kun Wu, Chen-Hsiang Lee, Keng-Liang Wu, Yi-Chun Chiu, Jing-Houng Wang, Sheng-Nan Lu, Seng-Kee Chuah

**Affiliations:** 1 Division of Hepato-gastroenterology, Department of Internal Medicine, Kaohsiung Chang Gung Memorial Hospital and Chang Gung University, College of Medicine, Kaohsiung, Taiwan; 2 Division of Infectious Disease, Department of Internal Medicine, Kaohsiung Chang Gung Memorial Hospital and Chang Gung University; College of Medicine, Kaohsiung, Taiwan; University of Aberdeen, United Kingdom

## Abstract

The influential roles of antibiotic prophylaxis on cirrhotic patients with peptic ulcer bleeding are still not well documented. The purpose of this study is to clarify these influential roles and to identify the risk factors associated with rebleeding, bacterial infection and in-hospital mortality. A cross-sectional, chart review study was conducted on 210 cirrhotic patients with acute peptic ulcer hemorrhage who underwent therapeutic endoscopic procedures. Patients were divided into group A (with prophylactic intravenous ceftriaxone, n = 74) and group B (without antibiotics, n = 136). The outcomes were length of hospital days, prevention of infection, rebleeding rate and in-hospital mortality. Our results showed that more patients suffered from rebleeding and infection in group B than group A (31.6% vs. 5.4%; p<0.001 and 25% vs. 10.8%; p = 0.014 respectively). The risk factors for rebleeding were active alcoholism, unit of blood transfusion, Rockall score, model for end-stage liver disease score and antibiotic prophylaxis. The risk factors for infection were active alcoholism, Child-Pugh C, Rockall score and antibiotic prophylaxis. Rockall score was the predictive factor for in-hospital mortality. In conclusions, antibiotic prophylaxis in cirrhotic patients after endoscopic interventions for acute peptic ulcer hemorrhage reduced infections and rebleeding rate but not in-hospital mortality. Rockall score was the predictive factor of in-hospital mortality.

## Introduction

Bacterial infection is a major problem in cirrhotic patients with upper gastrointestinal bleeding (UGIB). These patients frequently present infections at admission or develop them during hospitalization [1]–[3]. Bacterial infection is associated with an increased rate of failure to control bleeding [4], [5], rebleeding [5], [6] and plays a significant role in mortality [5], [7]. Antibiotic prophylaxis is considered a standard of care in these patients [7], [8].

Most published literatures focused on the characteristics and antibiotic prophylaxis in the cirrhotic patients with variceal bleeding [3], [9], [10], [11] or a mixture of nonvariceal and variceal bleeding analyzed together [1], [2]. Studies that clarify the effect antibiotic prophylaxis in cirrhotic patients with peptic ulcers bleeding alone are rare. Nonetheless, 30 to 40% of cirrhotic patients who bleed may have nonvariceal UGIB, and frequently caused by gastroduodenal ulcers [12]. Therefore, we conducted this study to clarify the influential roles of systemic antibiotic prophylaxis on cirrhotic patients with peptic ulcer bleeding after initial endoscopic treatments and identify the relevant risk factors. Intravenous ceftriaxone was selected for 2 reasons. First, administration of intravenous antibiotics is theoretically more appropriate than those administered orally in the prophylaxis of infection in patients with active UGIB. Second, it was reported that intravenous ceftriaxone is more effective than fluoroquinolone in areas of a high prevalence of quinolone-resistant organisms [13].

The end point of the study was to assess whether intravenous ceftriaxone is effective in reducing the rate of bacterial infections, occurrence of rebleeding and in-hospital mortality in cirrhotic patients with peptic ulcer hemorrhage after endoscopic interventions.

## Materials and Methods

### Ethics Statement

This retrospective chart review study was approved by both the Institutional Review Board and Ethics Committee of Chang Gung Memorial Hospital, Taiwan (IRB 103-0135B). All patients provided their written inform consent before endoscopic interventions. None of our patients belonged to the minors/children group.

### Patients

Between January 2007 and September 2013, a cross-sectional chart review study was conducted on 385 cirrhotic patients with peptic ulcer hemorrhage who underwent endoscopic interventions in Kaohsiung Chang Gung Memorial Hospital, Taiwan. A total of 210 patients who met the criteria were enrolled into current study (male/female: 159/51; mean age: 61.7±13.6 years) after strictly excluding those who did not meet the required criteria. The exclusion criteria were patients with existing signs of infection on admission (body temperature [BT]>38’C, white blood cell [WBC] count>10,000/ul, patients who received oral/parenteral antibiotics in the week prior to the procedure, the source of UGIB other than peptic ulcers hemorrhage, patients who died within the first day after admission or did not complete the in-hospital follow-up period were excluded for analysis of results. The enrolled patients were then divided into two groups: those who received prophylactic intravenous ceftriaxone (n = 74) and those who did not (n = 136). Those patients who did not receive antibiotics belonged to those occurred between January 2007 and December 2009 during which many physicians, including our hospital stuff over the emergency department, who were not aware of the issue of prescribing prophylactic antibiotics to these bleeding peptic ulcers in cirrhotic cohort which include our hospital stuff over the emergency department. It has been routine practice to prescribe prophylactic antibiotics to cirrhotic patients with gastrointestinal bleeding in our hospital since the year 2010 according to guidelines.

The antibiotic prophylaxis was given immediately after patients receiving endoscopic treatment and two sets of blood culture were obtained before administering the antibiotics according to our hospital practise. Gastric ulcers or duodenal ulcers bleeding was diagnosed by (1) clinical signs of hematemesis, coffee ground vomitus, hematochezia, or melena; (2) endoscopic signs of high-risk ulcers which were defined according to Forrest classification [14]. Timing from admission to the endoscopic treatment was measured and bleeding source was identified. Patients' statuses were stratified according to the Rockall classification [15]. All of our patients received endoscopic interventions within 24 hours on arrival at the emergency room and the endoscopic hemostasis were performed by experienced endoscopists. The registered clinical variables were demographic data, clinical manifestations of bleeding, and the use of tobacco, alcohol, aspirin, clopidogrel, non-steroidal anti-inflammatory drugs (NSAID), co-morbidities such as diabetes mellitus, cardiovascular disease, stroke, end-stage renal disease, and chronic pulmonary disease were registered. Other clinical characteristics such as age, sex, and hemodynamic instability on admission and laboratory data like white blood cells, hemoglobin, platelet count, prothrombin time, serum creatinine, serum albumin, and total bilirubin were analyzed. The end points were signs of infection, experienced rebleeding, length of hospital stay and death.

### Definitions

The diagnosis of cirrhosis was confirmed by clinical, laboratory, abdominal ultrasonographic, or histological findings [16]. The severity of cirrhosis was classified according to Pugh's modification of Child's classification [17]. The MELD score formula was 3.8×loge (Total bilirubin [mg/dL]) +11.2×loge (INR) +9.6×loge (creatinine [mg/dL]) +6.4×(etiology: 0 if cholestatic or alcoholic, 1 otherwise)[18]. The Child-Pugh score was according to the method described by Pugh et al [17].

Diagnosis of hepatitis C and B viruses-related liver disease was determined with specific viral markers (HBsAg or anti-HCV). Alcohol-related liver disease was defined as daily alcohol consumption >80 g in men and >40 g in women for at least 10 years with negative viral, metabolic, and autoimmune markers [19]. Active alcoholism is defined as a continuing daily alcohol intake over 20 g in patients with alcoholic cirrhosis [20].

Patients with peptic ulcer bleeding were treated with intravenous high dose pantoprazole (80 mg intravenously bolus followed by 200 mg continuous infusion for three days). Rebleeding was defined as a new onset of hematemesis, melena, or both associated with tachycardia or hypovolemic shock or a decrease in serum hemoglobin level of >2 g/dL after successful endoscopic and pharmacological treatment and hemodynamic stability of at least 24 hours period of stable vital signs [10]. Bleeding recurrence was confirmed by endoscopy in all cases.

Patients with infections were the total of patients with proven infections. Proven infection was defined when the diagnosis of bacteremia was made as the presence of viable bacteria in the blood and the clinical picture was consistent with this diagnosis.

The diagnosis of spontaneous bacterial peritonitis (SBP) was made when a positive culture of ascitic fluid was obtained with ascitic fluid neutrophils count > = 250 neutrophils/uL [21]. The diagnosis of pneumonia was made by clinical, radiological and bacteriological data. The diagnosis of urinary tract infection was made when a positive culture of urine (> = 10^5^ colonies/ml) was obtained with urine neutrophils count >10 neutrophils/uL and associated clinical pictures.

### Statistical Analysis

All results were expressed as means ± standard deviations for continuous variables and as relative frequencies or percentages for categorical variables. Distributions of continuous variables were analyzed by the X^2^ test, Fisher's exact test, or independent sample t test, depending upon the type of data analyzed for the two groups where appropriate. Kaplan–Meier analysis with the log-rank test was used to compare differences of rebleeding and death between the two groups. Variables were analyzed using multivariate Cox proportional hazard model to determine independent predictive factors of rebleeding and mortality. Only the variables that were significant in univariate analysis were analyzed in multivariate analysis. The results were expressed as odds ratios (OR) with 95% confidence intervals. To assess the prognostic utilities of the scoring systems, receiver operating characteristic (ROC) curves were plotted. The area under curve (AUC) was computed. An AUC over 0.7 was considered clinically useful, and that between 0.8 and 0.9 indicated an excellent diagnostic accuracy. An AUC of over 0.9 was seldom seen. All statistical analyses were performed using the SPSS v17.0 (Chicago, Illinois, USA). Statistical significance was taken as a p value <0.05.

## Results

### Demographic and clinical characteristics

The patients' demographics and clinical characteristics of the entire group and each of the cohorts are shown in Table 1. The two groups have comparable clinical and laboratory data except for longer prothrombin time in group A (13.8±5.1 vs. 12.4±2.8, p = 0.028). The mean age of patients was 61.7±13.6 years old with a male predominance (75.7%). The mean length of hospital stay was 14.4±11.3 days. Viral hepatitis was the most frequent etiology of cirrhosis (n = 146, 69.5%) followed by alcoholic (n = 57, 27.2%) and cryptogenic (n = 7, 3.3%). Child-Pugh's A, B and C patients account for 34.3%, 42.9%, 22.8% in study group. MELD and Child-Pugh score were 14.1±5.8 and 6.6±1.3 respectively.

**Table 1:** Clinical characteristics, endoscopic finding and clinical outcome of cirrhotic patients with peptic ulcer bleeding (n = 210).

Characteristics	Group A (n = 74)	Group B (n = 136)	P value
Age (years)	61.8±14.7	61.7±13.0	0.926
Male n (%)	59 (79.7)	100 (73.5)	0.317
Etiology of liver cirrhosis			
Alcoholic, n (%)	18 (24.3)	39 (28.7)	0.498
Viral hepatitis, n (%)	55 (74.3)	91 (66.9)	0.265
Cryptogenic, n (%)	1 (1.4)	6 (4.4)	0.238
Child-Pugh group			
A, n (%)	22 (29.7)	50 (36.8)	0.305
B, n (%)	36 (48.6)	54 (39.7)	0.252
C, n (%)	16 (21.6)	32 (23.5)	0.753
Rockall score	4.6±1.2	4.6±1.3	0.756
MELD score	14.6±5.8	13.9±5.8	0.426
Child-Pugh score	6.7±1.2	6.5±1.3	0.403
Ascites	31 (41.9)	52 (38.2)	0.605
Varices	46 (62.2)	80 (58.8)	0.637
EV	42	70	
IGV	1	1	
GOV	3	9	
Ulcer location			
Gastric ulcer, n (%)	48 (64.9)	85 (62.5)	0.734
Duodenal ulcer, n (%)	24 (32.4)	42 (30.9)	0.817
Both, n (%)	2 (2.7)	9 (6.6)	0.224
Use of NSAID or aspirin/clopidogrel, n (%)	12 (16.2)	17(12.5)	0.456
Smoking, n (%)	28 (37.8)	60 (44.1)	0.378
Active alcoholism, n (%)	23 (31.1)	53 (39.0)	0.256
Other comorbidities			
Diabetes mellitus, n (%)	27(36.5)	37(27.2)	0.163
Hypertension	33(44.6)	46 (33.8)	0.124
Cardiovascular disease, n (%)	2 (2.7)	7(5.1)	0.403
Stroke, n (%)	6 (8.1)	3(2.2)	0.070
ESRD, n (%)	4(5.4)	11(8.1)	0.471
COPD, n (%)	2 (2.7)	11 (8.1)	
Laboratory on admission			
WBC (10^9^/L)	6013.5±2039.9	5873.5±1967.0	0.627
Hb (g/dL)	8.7±2.4	8.9±2.0	0.522
PLT (10^9^/L)	112.0±51.4	120.6±71.0	0.313
Prothrombin time (s)	13.8±5.1	12.4±2.8	0.028
Albumin (g/dL)	2.9±0.7	2.8±0.7	0.516
Creatinine (mg/dL)	1.5±2.0	2.0±2.5	0.116
Total bilirubin (mg/dl)	3.1±3.5	2.9±3.6	0.751
Clinical characteristics			
Hypovolemic shock on admission, n (%)	6 (8.1)	12 (8.8)	0.860
Blood units transfused (unit)	4.9±6.1	5.4±8.0	0.714
Stigmata of recent hemorrhage at ulcer			
Forrest Ia or Ib ulcer, n (%)	35(47.3)	74 (54.4)	0.324
Forrest IIa or IIb ulcer, n (%)	36 (48.6)	53 (39.0)	0.175
Forrest IIc ulcer, n (%)	3 (4.1)	9 (6.6)	0.445
Time (h), bleeding to endoscopic treatment	7.5±8.6	7.8±8.0	0.754
Treatment			
Epinephrine injection	24 (32.4)	56 (41.2)	0.213
APC	12 (16.2)	29 (21.3)	0.372
Hemoclipping	7 (9.5)	6 (4.4)	0.270
Combined therapy	31 (41.9)	45 (33.1)	0.205
Epinephrine injection + APC	16 (21.6)	20 (14.7)	
Epinephrine injection + hemoclipping	15 (20.3)	25 (18.4)	
High dose PPI, n (%)	33 (44.6)	51 (37.5)	0.316
Outcomes			
Hospital stay (d)	16.3±12.4	13.4±10.6	0.092
Rebleeding, n (%)	4 (5.4)	43 (31.6)	<0.001
Infections, n (%)	8 (10.8)	34 (25.0)	0.014
In-hospital mortality, n (%)	11 (14.9)	26 (19.1)	0.440
Failure to control bleeding, n (%)	1 (1.4)	6 (4.4)	
Sepsis, n (%)	6 (8.1)	11 (8.1)	
Multiple organ failure, n (%)	4 (5.4)	9 (6.6)	

Abbreviations: APC, argon plasma coagulation; COPD, chronic obstructive pulmonary disease; ESRD, end-stage renal disease; EV, esophageal varices; GOV, Gastroesophageal varices; IGV, Isolated gastric varices; MELD, Model for End-Stage Liver Disease; NSAID, nonsteroidal anti-inflammatory drug; PPI, proton pump inhibitor; WBC, white blood cells; Hb, hemoglobin; PLT, platelet count; PT, prothrombin time.

Group A: antibiotic prophylaxis group.

Group B: control group.

One hundred and eighteen patients (56.2%) had concomitant co-morbidities other than cirrhosis itself while One hundred and twenty-nine patients (61.4%) had at least one risk factor associated with peptic ulcer diseases such as usage of Non-steroid anti-inflammation drugs (NSAID), anti-platelet agent, smoking and active alcohol consumption. Hemodynamic instability on admission was found in eighteen patients (8.6%).

### Endoscopic findings and Rockall scores

The mean time of patients receiving endoscopic intervention from admission was 7.7±8.2 hours. Gastric ulcers were the source of bleeding in 133 (63.3%) and duodenal ulcers in 66 (31.4%) patients. Concomitant bleeding gastric and duodenal ulcers occurred in 11 (5.3%) patients.

Most of these patients were found to have active ulcers with high stigmata of hemorrhage (Forrest Ia or Ib, 51.9% and Forrest II a or IIb, 43.3%). Among them, 60% was found to have varices (esophageal varices (EV): 53.3%; gastric varices (GV): 1%; combined EV/GV: 5.7%) but none of them have stigmata of recent hemorrhage (SRH). Mean value in the Rockall scoring system was 4.6±1.3 at admission, and 79.5% of patients had a value ≥4 (Score from 3 to 8).

### Medical and endoscopic treatment

The details of endoscopic interventions were summarized in table 1. Endoscopic intervention was performed in all patients with monotherapy with either (63.8%) or combination therapy (36.2%). All patients received intravenous proton pump inhibitors, either high-dose (40%) or non-high-dose intravenous PPI for 3 days (60%) after initial endoscopic hemostasis.

### Bacterial infections

**Figure 1:**
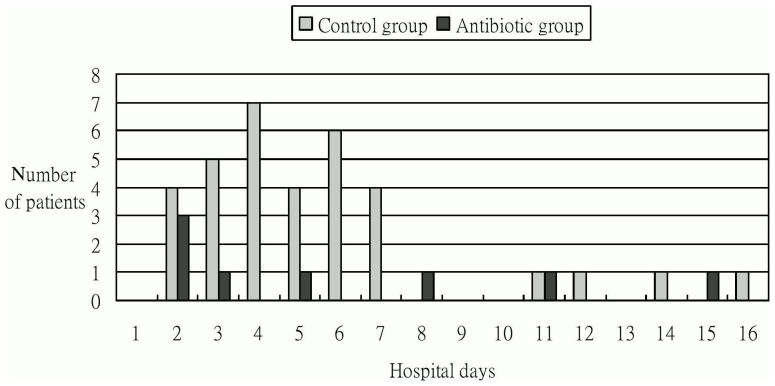
Distribution of the occurrence of bacterial infection (day 1 is the day of admission).

Overall, bacterial infections were documented in 42 patients (20%). Thirty-five of them (83.3%) were documented within 7 days of admission (Fig 1). More patients were infected in group B patients (10.8% vs. 25%; P = 0.014). Infections were confirmed for eight patients in group A (bacteremia in 4, spontaneous bacterial peritonitis in 1, and urinary tract infection in 3). On the other hand, thirty-four bacterial infections were proven in group B (bacteremia in 17, pneumonia in 4, spontaneous bacterial peritonitis in 4, and urinary tract infections in 9). The causative organisms of bacteremia are Gram-negative bacilli in fifteen patients (*Klebsiella pneumoniae* in 9; *Escherichia coli* in 6) and Gram-positive cocci in six patients (*Streptococcus pneumoniae* in 4; *Staphylococcus aureus* in 1; *Enterococcus faecalis* in 1).

Multivariate analysis identified three independent predictors for bacterial infection such as active alcoholism (OR: 2.229; 95% CI = 1.196–4.154; P = 0.012), Child-Pugh's class C (OR: 1.980; 95% CI = 1.056–3.713; P = 0.033), Rockall score (OR: 1.363; 95% CI = 1.094–1.699; P = 0.006) and antibiotic prophylaxis (OR: 0.391; 95% CI: 0.179–0.855; P = 0.019), (Table2).

**Table 2:** Univariate and Multivariate Analysis of Potential Risk Factors for infection in Patients with peptic ulcer Bleeding Following Endoscopic Treatment.

Variable	Univariate analysis		Multivariate analysis	
	Odds ratio (95% CI)	*P* value	Odds ratio (95% CI)	*P* value
Age	0.989 (0.967–1.010)	0.303		
Male gender	1.231 (0.589–2.575)	0.580		
Etiology of liver cirrhosis				
Alcoholic	1.692 (0.888–3.221)	0.110		
Viral hepatitis	0.608 (0.326–1.136)	0.119		
Cryptogenic	1.082 (0.261–4.478)	0.914		
Child-Pugh class C	2.151 (1.156–4.002)	0.016	1.980 (1.056–3.713)	0.033
Varices	1.489 (0.774–2.865)	0.225		
Ascites	0.694 (0.365–1.320)	0.265		
Other comorbidities				
Diabetes mellitus	1.136 (0.598–2.159)	0.697		
CVD	1.326 (0.182–9.686)	0.781		
Stroke	0.575 (0.079–4.186)	0.585		
ESRD	0.628 (0.152–2.604)	0.522		
COPD	1.892 (0.740–4.832)	0.183		
Use of NSAID or aspirin	0.147 (0.020–1.069)	0.058		
Smoking	1.138 (0.617–2.098)	0.679		
Active Alcoholism	1.893 (1.031–3.477)	0.039	2.229 (1.196–4.154)	0.012
WBC (10^9^/L)	1	0.719		
Hb (g/dL)	0.904 (0.786–1.039)	0.156		
PLT (10^9^/L)	0.995 (0.989–1.001)	0.084		
PT (second)	1.309 (0.789–2.170)	0.297		
Creatinine (mg/dL)	0.977 (0.849–1.124)	0.744		
Albumin (g/L)	0.902 (0.564–1.442)	0.666		
Total bilirubin	1.074 (1.019–1.132)	0.008		
Blood units transfused	1.016 (0.992–1.041)	0.193		
Hypovolemic shock on admission	1.227 (0.438–3.438)	0.698		
Time (h), bleeding to endoscopic treatment	1.022 (0.991–1.055)	0.170		
Stigmata of recent hemorrhage				
Forrest Ia or Ib	0.655 (0.355–1.207)	0.175		
Forrest IIa or IIb	1.642 (0.894–3.017)	0.110’		
Forrest IIc	0.558 (0.077–4.065)	0.565		
Rockall score	1.361 (1.093–1.695)	0.006	1.363 (1.094–1.699)	0.006
MELD score	1.046 (1.002–1.092)	0.039		
Child-Pugh score	1.231 (0.993–1.527)	0.058		
Recurrent bleeding	2.198 (1.179–4.100)	0.013		
Antibiotic prophylaxis	0.340 (0.156–0.738)	0.006	0.391 (0.179–0.855)	0.019

Abbreviations: CI, confidence interval; COPD, chronic obstructive pulmonary disease; CVD, Cardiovascular disease; ESRD, end-stage renal disease; MELD, Model for End-Stage Liver Disease;, NSAID, nonsteroidal anti-inflammatory drug; PPI, proton pump inhibitor; WBC, white blood cells; Hb, hemoglobin; PLT, platelet count; PT, prothrombin time.

### Rebeeding and mortality

Overall in-hospital mortality occurred in 37 (17.6%) patients while 47 (22.4%) patients had rebleeding. MELD, Rockall and Child-Pugh's score were all found to have good predictive value for in-hospital mortality (all p-value<0.001 and area under curve AUC: 0.748, 0.782, 0.683 respectively)

**Figure 2:**
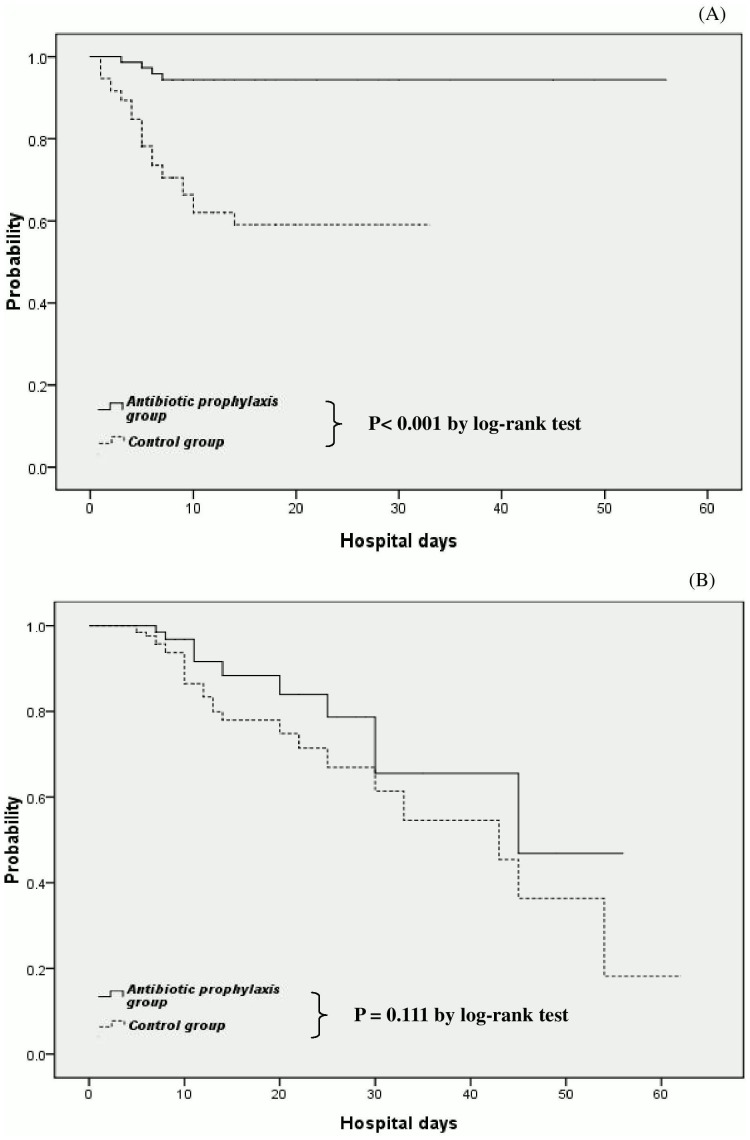
Actuarial probability of remaining free of rebleeding in all cirrhotic patients after endoscopic interventions for ceftriaxone group (antibiotic prophylaxis group) and the no antibiotic prophylaxis group (control group) (P<0.001 by log-rank test) (A). Actuarial probability of remaining survival in all cirrhotic patients after endoscopic interventions for ceftriaxone group (antibiotic prophylaxis group) and the no antibiotic prophylaxis group (control group) (P = 0.111 by log-rank test)(B).

There were more rebleeding events in group B than group A (31.6% vs. 5.4%, p<0.001)(Table 1). When we analyzed the hemostatic outcome of the two groups of patients, the actuarial probability of remaining free of rebleeding in hospital after endoscopic therapy among patients with peptic ulcer bleeding was significantly lower in group A (P<0.001 by log-rank test) (Figure 2A).

Causes of death were uncontrolled bleeding in 7 patients (1 in the ceftriaxone group and 6 in the control group), multiple organ failure in 13 patients (4 and 9 patients, respectively), and sepsis in 17 patients (6 and 11 patients, respectively) (Table 1). There was no significant difference in mortality during hospitalization after inclusion between patients treated with intravenous ceftriaxone (n = 11, 14.9%) and those treated without antibiotic prophylaxis (n = 26, 19.1%). The observed survival was virtually identical for both groups (P = 0.111 by log-rank test) (Figure 2B).

**Figure 3:**
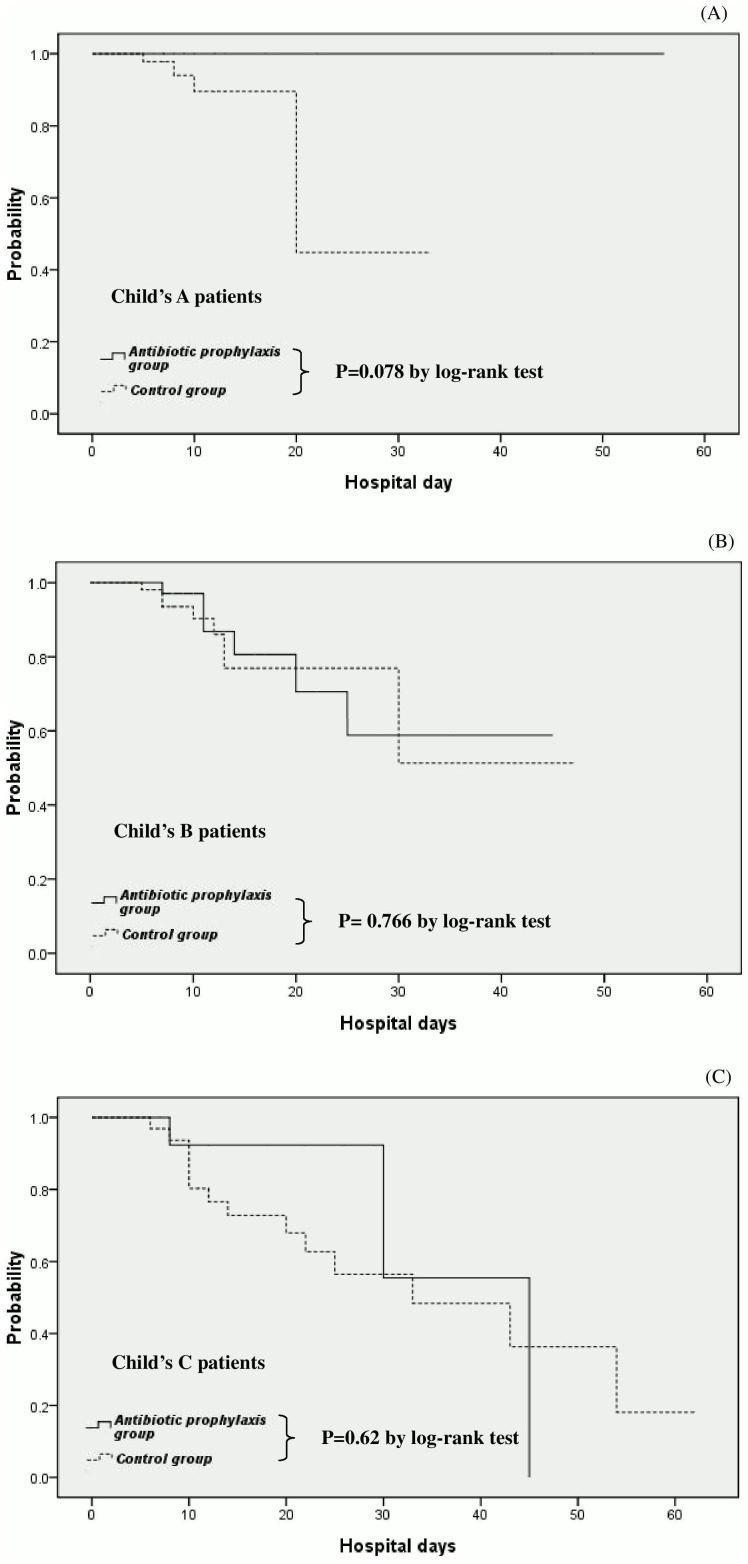
Actuarial probability of remaining survival at different clinical stages of cirrhotic patients after endoscopic interventions for ceftriaxone group (antibiotic prophylaxis group) and the no antibiotic prophylaxis group (control group). There was a similar probability of remaining survival between patients who were prescribed with intravenous ceftriaxone and those without antibiotic prophylaxis in Child's A, B and C group (P = 0.078, 0.766 and 0.620 by log-rank test, respectively) (A, B and C).

However, when we performed subgroup analysis according to different clinical stages of cirrhotic patients, there was a similar probability of remaining survival between patients who were prescribed with intravenous ceftriaxone and those without antibiotic prophylaxis in Child's A, B and C group (p = 0.078, 0.766 and 0.620 by log-rank test, respectively) (Figure 3 A, B and C).

### Independent risk factors for rebleeding and death

The results of the univariate and multivariate analysis for independent risks of rebleeding and death were summarized in Tables 3 and 4 respectively. An increased risk of rebleeding after endoscopic hemostasis was associated with active alcoholism (OR = 1.923; 95% CI: 1.060–3.491, p = 0.031), blood unit transfusion (OR = 1.024; 95% CI: 1.004–1.004, p = 0.017), Rockall score (OR = 1.063; 95% CI: 1.004–1.125, p = 0.037), MELD score (OR = 1.119; 95% CI: 1.049–1.194, p = 0.001) and antibiotic prophylaxis (OR = 0.122, 95% CI: 0.043–0.344, p<0.001) (Table 3). The results of univariate analysis showed that male gender, total bilirubin level, Rockall score, MELD score, Child-Pugh score, bacterial infection and recurrent bleeding were associated with an increased risk of death. However, according to the results of multivariate analysis, the independent predictors of in-hospital death were only Rockall score (OR = 1.809; 95% CI: 1.419–2.306, p<0.001) (Table 4).

**Table 3:** Univariate and Multivariate Analysis of Potential Risk Factors for Rebleeding in Patients With peptic ulcer Bleeding Following Endoscopic Treatment.

Variable	Univariate analysis		Multivariate analysis	
	Odds ratio (95% CI)	*P* value	Odds ratio (95% CI)	*P* value
Age	0.993 (0.973–1.013)	0.493		
Male gender	3.049 (1.205–7.711)	0.019		
Etiology of liver cirrhosis				
Alcoholic	1.203 (0.635–2.279)	0.571		
Viral hepatitis	0.968 (0.518–1.809)	0.918		
Cryptogenic	0.414 (0.057–3.004)	0.383		
Child-Pugh class C	2.278 (1.562–4.940)	<0.001		
Varices	1.486 (0.637–3.468)	0.359		
Ascites	0.687 (0.372–1.269)	0.231		
Other comorbidities				
Diabetes mellitus	1.255 (0.692–2.277)	0.454		
CVD	0.917 (0.125–6.733)	0.932		
Stroke	1.495 (0.362–6.176)	0.578		
ESRD	1.284 (0.460–3.581)	0.633		
COPD	0.043 (<0.001–4.753)	0.190		
Use of NSAID or aspirin	0.718 (0.284–1.816)	0.484		
Smoking	1.373 (0.774–2.436)	0.279		
Active alcoholism	1.773 (1-3.142)	0.050	1.923 (1.060–3.491)	0.031
WBC (10^9^/L)	1	0.510		
Hb (g/dL)	0.980 (0.860–1.118)	0.765		
PLT (10^9^/L)	1 (0.995–1.004)	0.954		
PT (second)	1.242 (0.715–2.157)	0.442		
Creatinine (mg/dL)	1.076 (0.982–1.178)	0.115		
Albumin (g/L)	0.654 (0.410–1.044)	0.075		
Total bilirubin	1.104 (1.041–1.170)	0.001		
Blood units transfused	1.030 (1.013–1.047)	0.001	1.024 (1.004–1.044)	0.017
Hypovolemic shock on admission	1.081 (0.388–3.012)	0.882		
Time (h), bleeding to endoscopic treatment	1.003 (0.970–1.037)	0.852		
Stigmata of recent hemorrhage				
Forrest Ia or Ib	1.325 (0.740–2.374)	0.343		
Forrest IIa or IIb	0.797 (0.443–1.436)	0.450		
Forrest IIc	0.586 (0.081–4.258)	0.598		
Rockall score	1.265 (1.017–1.573)	0.035	1.063 (1.004–1.125)	0.037
MELD score	1.069 (1.026–1.115)	0.001	1.119 (1.049–1.194)	0.001
Child-Pugh score	1.391 (1.135–1.703)	0.001		
High dose PPI	0.834 (0.469–1.483)	0.537		
Combined treatment of endoscopic hemostasis	0.951 (0.520–1.739)	0.871		
Bacterial infection	2.073 (1.133–3.793)	0.018		
Antibiotic prophylaxis	0.129 (0.046–0.360)	<0.001	0.122 (0.043–0.344)	<0.001

Abbreviations: CI, confidence interval; COPD, chronic obstructive pulmonary disease; CVD, Cardiovascular disease; ESRD, end-stage renal disease; MELD, Model for End-Stage Liver Disease; NSAID, nonsteroidal anti-inflammatory drug; PPI, proton pump inhibitor; WBC, white blood cells; Hb, hemoglobin; PLT, platelet count; PT, prothrombin time.

**Table 4:** Univariate and Multivariate Analysis of Potential Risk Factors for Death in Patients with peptic ulcer Bleeding Following Endoscopic Treatment.

Variable	Univariate analysis		Multivariate analysis	
	Odds ratio (95% CI)	*P* value	Odds ratio (95% CI)	*P* value
Age	0.989 (0.965–1.013)	0.352		
Male gender	2.694 (1.038–6.989)	0.042		
Etiology of liver cirrhosis				
Alcoholic	1.079 (0.508–2.292)	0.844		
Viral hepatitis	0.945 (0.456–1.959)	0.880		
Cryptogenic	0.905 (0.122–6.690)	0.922		
Child-Pugh class C	1.703 (0.879–3.302)	0.115		
Varices	1.644 (0.774–3.493)	0.196		
Ascites	0.587 (0.294–1.174)	0.132		
Other comorbidities				
Diabetes mellitus	0.983 (0.496–1.947)	0.961		
CVD	1.356 (0.307–5.992)	0.688		
Stroke	1.125 (0.259–4.880)	0.875		
ESRD	1.638 (0.497–5.402)	0.417		
COPD	0.680 (0.163–2.845)	0.598		
Use of NSAID or aspirin	0.593 (0.182–1.934)	0.386		
Smoking	0.837 (0.420–1.669)	0.614		
Active alcoholism	1.749 (0.914–3.346)	0.091		
WBC (10^9^/L)	1	0.118		
Hb (g/dL)	0.900 (0.768–1.055)	0.193		
PLT (10^9^/L)	1 (0.994–1.005)	0.881		
PT (second)	1.317 (0.843–2.247)	0.201		
Creatinine (mg/dL)	1.060 (0.924–1.216)	0.403		
Albumin (g/L)	0.865 (0.508–1.474)	0.594		
Total bilirubin	1.090 (1.035–1.147)	0.001		
Blood units transfused	0.990 (0.961–1.019)	0.486		
Hypovolemic shock on admission	1.613 (0.568–4.576)	0.369		
Time (h), bleeding to endoscopic treatment	0.981 (0.944–1.020)	0.331		
Stigmata of recent hemorrhage				
Forrest Ia or Ib	0.998 (0.519–1.918)	0.994		
Forrest IIa or IIb	0.881 (0.452–1.716)	0.709		
Forrest IIc	2.405 (0.571–10.134)	0.232		
Rockall score	1.805 (1.414–2.304)	<0.001	1.809 (1.419–2.306)	<0.001
MELD score	1.071 (1.022–1.122)	0.004		
Child-Pugh score	1.342 (1.079–1.669)	0.008		
High dose PPI	0.562 (0.290–1.091)	0.089		
Combined treatment of endoscopic hemostasis	0.730 (0.376–1.416)	0.352		
Bacterial infection	1.939 (1.006–3.738)	0.048		
Recurrent bleeding	2.534 (1.307–4.914)	0.006		
Antibiotic prophylaxis	0.570 (0.280–1.162)	0.122		

Abbreviations: CI, confidence interval; COPD, chronic obstructive pulmonary disease; CVD, Cardiovascular disease; ESRD, end-stage renal disease; MELD, Model for End-Stage Liver Disease; NSAID, nonsteroidal anti-inflammatory drug; PPI, proton pump inhibitor; WBC, white blood cells; Hb, hemoglobin; PLT, platelet count; PT, prothrombin time.

## Discussion

Bacterial infection is a frequent complication in cirrhotic patients with upper gastrointestinal bleeding and may account for severe complication and mortality [1], [2]. It is associated with failure to control bleeding and patients with recurrent bleeding episodes [5], [6]. The role of intestinal bacteria as a major source of infection in cirrhotic patients may explain why prophylactic antibiotics are so effective in reducing infections during gastrointestinal hemorrhage. Therefore, it is rational that prescribing broad-spectrum antibiotic prophylaxis at bleeding presentation may improve survival and reduce sepsis complications [1], [2]. Currently, antibiotic prophylaxis is standard care of cirrhotic patients with acute variceal bleeding but the influential roles of antibiotic prophylaxis on cirrhotic patients with peptic ulcer bleeding are still not well documented.

The results of current study proved the benefit of antibiotic prophylaxis for bleeding peptic ulcer in cirrhotic patients with only 8 of the 74 patients (10.8%) included in group A developed bacterial infections after hospitalization. On the other hand 34 of the 136 patients (25%) without antibiotic prophylaxis suffered more infections (P<0.014) in group B. This was similar to other studies [1]–[3], showing that he antibiotic prophylaxis could reduce the infection rate of cirrhotic patients with peptic ulcer bleeding. Our study added that antibiotic prophylaxis in cirrhotic patients with bleeding peptic ulcers was could reduce the rebleeding events but did not reduce in-hospital mortality or shorten length of hospitalization.

The incidence of bacterial infection in our study (10.8% in group A and 25% in group B) was much lower than those in other reports [1]–[3]. The discrepancy could be due to the differences in the designs of the studies. First, patients who already had been infected upon arrival were excluded, because bacterial infection in cirrhotic patients with upper gastrointestinal bleeding usually occurs early before admission [1], [7]. Most patients (83.3%) developed bacterial infections within 7days of admission. Second, our patients had better liver functional reserve. It had been documented that bacterial infections were more frequent in Child-Pugh's C patients than in those with Child-Pugh's A or B [22]. There were only 22.8% of Child-Pugh C patients in our study cohort in contrast with 71% in the study of Pauwels *et al* [2] and 80% in the study of Blaise *et al* [3]. Third, alcoholic cirrhosis is prone to infection but not chronic active hepatitis [22], [23]. The etiology of cirrhosis in our study cohort was mainly due to hepatitis B or C (69.5%) as opposed to alcohol in previous studies [1]–[3].

The rebleeding rate observed in current study (22.3%) was higher than those reported in non-cirrhotic patients (3.2%) [24]. The higher frequency of rebleeding may be partly due to more high risks ulcers (Forrest Ia, Ib: 51.9% and Forrest IIa, IIb: 42.4%) were found in all of our patients. On the other hand, 60% of patients were found to have varices, 65.7% with advanced stage of cirrhosis (Child-Pugh's class B or C) indicating liver decompensation and probably existence of bleeding tendency.

Active alcoholism is independent predictors of both adverse outcomes as in-hospital rebleeding and bacterial infection. Both acute and chronic alcohol use can affect the immune system at the level of innate or acquired immune response. Altered inflammatory neutrophil, leukocyte, and macrophage functions after alcohol consumption contribute to impaired host defense against microbial infections [25]. In addition, active alcoholism can increase host susceptibility to bacterial infection, especially in cirrhotic patients with immunocompromised status [22], [23].

Our study result released an important message that the efficacy of intravenous ceftriaxone in the reducing mortality in cirrhotic patients with peptic ulcer hemorrhage is relatively poor. Overall, eleven of the 74 patients (14.9%) treated with antibiotic prophylaxis died during hospitalization and the major cause of mortality was sepsis. In our study, in-hospital mortality was significantly higher than that reported in non-cirrhotic or cirrhotic patients from three studies (17.6 vs. 5.4, 4.5 and 13.8%) [24]. There was only 21% of bleeding-related mortality and the rest died of severe sepsis and multiple organ failure suggesting that concomitant co-morbidities had a fundamental role in the occurrence of death. Based on the results of our study, predictors of in-hospital death in cirrhotic patients with peptic ulcer bleeding proved to be substantially different from those observed in non-cirrhotics.

Rockall score were found to be independent predictors of in-hospital mortality. Rockall scoring system was based on multivariate analysis of information from history, examination, blood tests, and endoscopic investigation. It makes use of both clinical and endoscopic criteria to predict the risks of rebleeding and death; the scale ranges from 0 to 11 points, with higher scores indicating higher risk. Therefore, the Rockall risk assessment score was devised to allow prediction of the risk of rebleeding and death in patients with acute non-variceal upper gastrointestinal hemorrhage. In the present study, we used Rockall's risk scoring system to classify patients and found that high clinical Rockall scores were associated with adverse outcomes (rebleeding, bacterial infection and death), and the results obtained were widely corroborated in clinical practice.

Among the 47 (22.4%) patients who encountered rebleeding, 7 (3.3%) patients died of uncontrolled bleeding. The Rockall score was found to be significantly higher than the survivors (5.4±1.6 VS 4.3±1.1, P = 0.016). For mortality analysis, the area under the ROC curve was 0.782 (95%CI: 0.691–0.872, *P*<0.001). These were consistent with other studies suggesting that the Rockall score had a good predictive value for in-hospital mortality [26], [27].

We also observed that there is no difference in use of high dose PPI to reduce the risk of rebleeding. This may be explained the reduced acid secretion in patients with liver cirrhosis [28]. In fact, the evidences of the efficacy of high dose PPIs for bleeding ulcers in cirrhotic patients is still limited. It is therefore very important to target for an appropriate endoscopic hemostasis and antibiotic prophylaxis as a complement to PPIs in cirrhotic patients with high-risk bleeding stigmata at endoscopy.

Current study encounters some limitations. First, this was a single center report and the sample size is relatively small so bias may exist and caution must be taken in extrapolating the results. A multicenter data with large sample size is mandatory. Second, this is a retrospective chart review study with observations based on hospitalized patients with upper gastrointestinal bleeding.

## Conclusions

This current study enhances the evidences of beneficial effects of antibiotic prophylaxis in patients with cirrhosis with peptic ulcer bleeding. Antibiotic prophylaxis in cirrhotic patients after endoscopic interventions for acute peptic ulcer hemorrhage reduced infections and rebleeding rate but not in-hospital mortality. Rockall score was the predictive factor of in-hospital mortality. A majority of cirrhotic patients with peptic ulcer bleeding in hospital died of non-bleeding-related causes, for example, sepsis and multiple organ failure. This calls for an energetic effort for the use of antibiotic prophylaxis and a stronger support of other major organ systems in these patients cohort. Further studies should be directed to explore ways to improve the overall outcome of the patients.
